# Using Artificial Intelligence for Pattern Recognition in a Sports Context

**DOI:** 10.3390/s20113040

**Published:** 2020-05-27

**Authors:** Ana Cristina Nunes Rodrigues, Alexandre Santos Pereira, Rui Manuel Sousa Mendes, André Gonçalves Araújo, Micael Santos Couceiro, António José Figueiredo

**Affiliations:** 1 Coimbra Polytechnic, Instituto Superior de Engenharia de Coimbra, 3030-199 Coimbra, Portugal; 2 Faculdade de Ciência e Tecnologia, New University of Lisbon, 2829-516 Lisbon, Portugal; als.pereira@campus.fct.unl.pt; 3 Coimbra Polytechnic, Escola Superior de Educação de Coimbra, 3030-329 Coimbra, Portugal; rmendes@esec.pt; 4 Ingeniarius Lda, 3025-307 Coimbra, Portugal; andre@ingeniarius.pt (A.G.A.); micael@ingeniarius.pt (M.S.C.); 5 Univ Coimbra, Research Unit for Sport and Physical Activity, Faculty of Sport Sciences and Physical Education, 3040-248 Coimbra, Portugal; afigueiredo@fcdef.uc.pt

**Keywords:** artificial intelligence, artificial neural network, long short-term memory, ensemble classification method, wearable technology, sports

## Abstract

Optimizing athlete’s performance is one of the most important and challenging aspects of coaching. Physiological and positional data, often acquired using wearable devices, have been useful to identify patterns, thus leading to a better understanding of the game and, consequently, providing the opportunity to improve the athletic performance. Even though there is a panoply of research in pattern recognition, there is a gap when it comes to non-controlled environments, as during sports training and competition. This research paper combines the use of physiological and positional data as sequential features of different artificial intelligence approaches for action recognition in a real match context, adopting futsal as its case study. The traditional artificial neural networks (ANN) is compared with a deep learning method, Long Short-Term Memory Network, and also with the Dynamic Bayesian Mixture Model, which is an ensemble classification method. The methods were used to process all data sequences, which allowed to determine, based on the balance between precision and recall, that Dynamic Bayesian Mixture Model presents a superior performance, with an F1 score of 80.54% against the 33.31% achieved by the Long Short-Term Memory Network and 14.74% achieved by ANN.

## 1. Introduction

Pattern recognition is the ability of a machine to recognize patterns in their environment, using artificial intelligence learning abilities to make decisions. The whole process involves the extraction of information from sensory signals, wherein wearable technology deserves all the prominence when it comes to having a system capable of analyzing and predicting the human movement [1]. Several works have compared the performance of wearable-based systems with professional laboratory/clinic equipment and verified that, while results were rather similar, wearable solutions were additionally able to keep the ecological validity of task [2]. This implies that the data extracted with wearable solutions shall represent ecological patterns of the human movement inherent to various areas of application, such as sports, speech and face recognition, classification of handwritten characters, medical diagnosis and rehabilitation [3].

Wearable technologies allow to extract, on-the-fly and in a real-world context, kinematic variables, such as the position, velocity, orientation, and others, and physiological data, such as electromyography (EMG) and heart rate. Yet, it is still unclear how this large amount of data can be translated into simple and practical data. This is considerably more challenging in the context of sports, where coaches, sports analysts, exercise physiologists and athletes need to have access to this data in real-time, to improve the understanding of the athlete’s behaviour during training and competition [4].

In collective sports, such as futsal, athletes can run for several kilometres during a single match, adopting different types of locomotion: moving on a straight line, side run and quick changes of direction, in conjunction with different velocities and rhythms, revealing how collective sports can be challenging for pattern recognition [5,6,7]. The literature shows that, for a complete analysis of the gait and running pattern, as with other related actions, one should assess a wide range of data on-the-fly. Clermont et al. [8] proposed a model to identify sex-specific groups of both competitive or recreational runners based on the centre of mass accelerations. To do that the authors used one of the most traditional machine learning method, the Support Vector Machine (SVM), using 24 features extracted from accelerometers. The SVM led to a classification of 82.63% and 80.4% for competitive and recreational runners in the male and female models, respectively, emphasizing the fact that competitive runners exhibited a more consistent running gait pattern. Despite the results, the authors shed no light on the impact and the methodology adopted to select the most appropriate features, being currently one of the major gaps in the literature. According to Yang et al. [9], no predefined features had yet been found that could be employed to better distinguish and classify different activities. To solve that problem, Yang et al. proposed and architecture that used Convolutional Neural Networks (CNN) to capture salient sensory data patterns at different time scales. Then, the data patterns were consolidated between channels and subsequently mapped into different classes depending on the activities in the study. An interesting and differential factor about this methodology was that feature extraction was not accomplished by hand, thus reducing the error and providing a better discriminating power. More recently, Zhu et al. [10] proposed an action recognition algorithm based on several features and a modified deep learning method using video data. For the pre-training process, the authors used the Restricted Boltzmann Machine (RBM) to learn and optimize parameters. Then, using deep learning, they constructed the deep beliefs nets (DBN), composed of various RBMs in series, in which the most interesting points out of 13 human joints were extracted. At the end of the process, the authors used an SVM to fine-tune the DBN network, concluding that, with this approach, it was possible to reduce the number of samples to train the model, thus reducing the training process and achieving an averaged accuracy of 98%. By also exploring the benefits of ensemble classifiers, Vital et al. [11] described how a set of human movements in daily activities can be recognized using a wearable motion capture suit and a Dynamic Bayesian Mixture Model (DBMM). The DBMM relies on the computed confidence belief from base classifiers, including SVM, Artificial Neural Networks (ANN), and others, and combining their likelihoods into a single result. Conforti et al. [12] worked on a solution for the recognition of postural patterns using wearable sensors and machine-learning algorithms, using kinematic data as input. The participants were asked to performed tasks, such as lifting and releasing small loads, with a correct and incorrect posture. The train and test were done with the SVM model, leading to a 76.9% accuracy when fed by raw data and 99.4% using features computed from this raw data, proving the susceptibility of the SVM to selected features. Still around action recognition, Huang et al. [13] designed a pre-processing method for a 3-axis accelerometer to remove the noise and a combined Kalman and genetic algorithm, which was used to encode Kalman’s filter parameters. They used a threshold, determined by the cross-validation method, and eigenvalues to distinguish falls from normal daily activities. There were eleven target actions and fifteen types of falls. The results showed that fall recognition accuracy is better than others in the literature, presenting a recall rate of 99.1%.

Extending the domain of application to sports, to get more information about the athletes promoting a healthier lifestyle and improving performance, has also been trendy [14]. The most common research in this domain employs kinematic data acquired using cameras or more recently, wearables. The use of wearable technology in sports has increased in the past years [15], although the results achieved so far are still far behind the expectations [16]. Rossi et al. [17] proposed a multi-dimensional approach to predict and prevent injuries in football players. The authors used GPS measurements to feed a machine learning method which showed good accurate and interpretable results. Grunz et al. [18] proposed a method to detect different tactical patterns by comparing data from different players using ANN. Montoliu et al.’s work [19] showed the application of pattern recognition concepts and the bag-of-words technique using football videos and testing several classifiers. The authors were able to prove the ability of the approach to recognize some specific actions, as ball possession. Some authors have additionally focused on physiological data, as it is the case of Boca and Park [20], who proposed an ANN architecture to recognize actions using myoelectric data. The fuzzy c-means algorithm was adopted and combined with a Fourier analysis for feature extraction. Another ANN approach was proposed by Al-Mulla and Sepulveda [21], whose method performed both filtering and signal classification. Five inputs were considered for training and one for testing and the results showed an average prediction inaccuracy of 9.2%.

With all these works in mind, the main objective of this study, besides implying the multisensorial fusion for data acquisition, is threefold:

1. Investigate the feasibility of combining wearable technologies, namely *Myontec*’s MBody3 and *Ingeniarius*’s TraXports, to recognize actions performed by athletes, taking into consideration their bio-signals, position and orientation over time.
2. Evaluate which are the most adequate features for the identification of multiple futsal actions, namely (i) running; (ii) running with the ball; (iii) walking; (iv) walking with the ball; (v) passing; (vi) shooting; and (vii) jumping.
3. Compare the performance of two classification algorithms: a type of Recurrent Neural Network (RNN), known as Long Short-term Memory (LSTM) network, and an ensemble classification method, know as Dynamic Bayesian Mixture Model (DBMM).

For several years, football equipment that allows to collect data from the field have been made available worldwide. If the possibility to analyse this data in real-time was presented, it would be possible to correct the slightest error during competitions. Until today, most of this data is collected based on visual analysis using cameras. This data can be statistical (i.e., the number of passes or shots of a given player or the whole team), and positional, from where it is possible to estimate the distances covered by athletes. With a system based on wearable technology, like the one presented here, we have the possibility of acquiring all these data on-the-fly and with greater accuracy, since it is not affected by light variations nor occlusions such as cameras.

This study aims to provide possibilities for coaches and technical teams to analyse team performance, which is key to achieve better outcomes. Performance analysis in this article is achieved by analysing physiological and kinematic data in a holistic approach through an innovative solution, thus representing a differential advantage from current alternatives available in the market that focus entirely on positional data. Moreover, by being in direct contact with the athletes’ body, these solutions can go beyond performance analysis, with strong applicability in injury prevention by acquiring and analysing physiological data. However, all these technologies have been harnessing the Big Data phenomenon, leading to a “datafication” of the data, wherein team sports coach seem unable to adequately interpret the meaning of large and complex volumes of data, being then unfeasible to implement sustainable solutions that may be used to enhance performance. Therefore, these technologies need to go hand-in-hand with scientific advances. This paper paves the way towards the development of approaches able to support sport scientists on understanding athlete and team performance during competition and training. The paper presents a methodology and related benchmark with the end-goal of providing relevant information to practitioners and administrators in sports programs, with the intent to move from a data-driven to a data-informed focus, combining the power of new technologies and sports science to improve athletic performance. When performing a study that portrays as much as possible what happens in the field in real-time, where it is assumed that the data used is not treated in such a way as to have all the requirements that could classify them as optimal data, it must be assumed that the results obtained do not present the best possible solutions to be applied in high competition. However, in our eyes, this is a study with enormous potential and we believe it is capable of arousing the interest of more authors in this area.

This paper is divided as follows: Section 2 presents the methodology adopted, including (i) a description of the experimental setup, the wearable devices and their ecological value in sports, (ii) data extracted, (iii) data analysis framework which includes feature selection. Lastly, it is described the classification methods adopted and compared in this study for human movement recognition. Section 3 encompasses the experimental results. Section 4 describes the conclusions and also some considerations for future work.

## 2. Methodology

### 2.1. Description of Futsal Players and Matches Analysed

The main aim of this research paper is to recognize the athlete’s actions as identified in Table 1.

**Figure 1:**
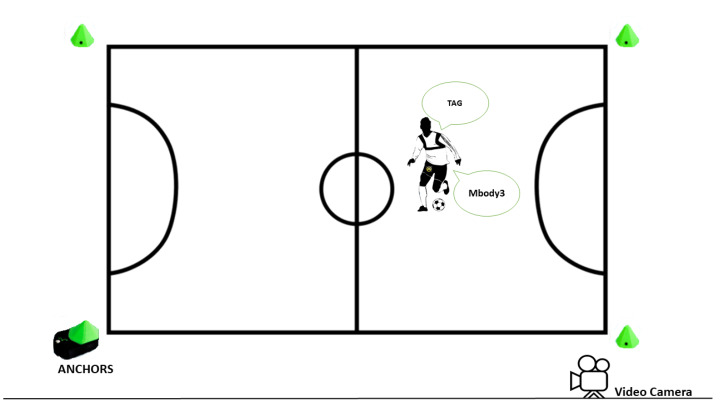
Representative Illustration of the setup.

Twenty-two injury-free males (22.2 ± 4.5 years, with max 39 years and min 19 years) have participated in this study. The study was approved by the Ethics Committee of the Polytechnic Institute of Coimbra (14 CEIPC2/2019). The study consisted of recording four futsal matches divided in a two-day tournament. Each of the four games had two-parts with 10 min per part. Before the beginning of the games, the four UWB anchors of the TraXports system were deployed in the field, as illustrated in Figure 1.

All the players wore the TraXports, though only one of the individuals wore the Mbody3. That player, tagged as number 10, provided the trigger to initialize the system, ensuring that both positional and physiological data are simultaneously tracked.

**Figure 2:**
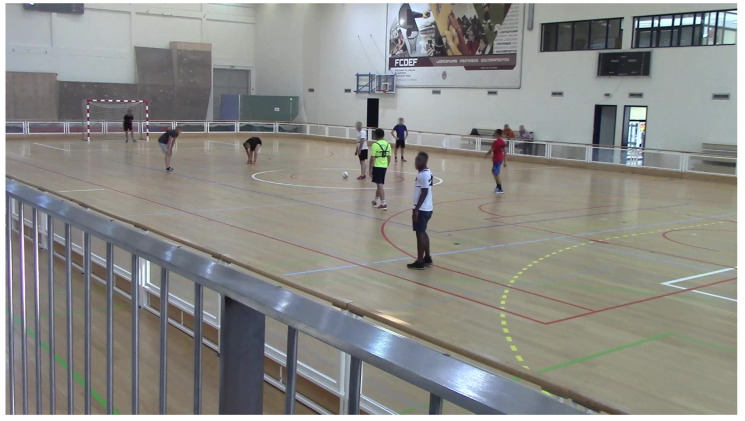
A game frame acquired by the video camera.

Allied to that, and for a posterior ground truth analysis, we placed a video camera with next to the benches, where it was possible to keep track of all the players in the field as it can be seen in Figure 2. The beginning of the videos matched with the beginning of the match. The trials were labelled by hand. Therefore, such synchronization between wearables and videos was necessary to adequately label the trials.

### 2.2. Description of the Instruments Used

This study used MATLAB for processing data collected by both TraXports and MBody3, as well as to build the architecture to classify the data. The DBMM framework developed by Faria et al. [22] and further improved by [11] was adopted. The LSTM network was implemented using MATLAB’s *Deep Learning Toolbox* based on [23].

TraXports and MBody3 are the acquisition instruments considered in this study and categorized as wearable devices.

**Figure 3:**
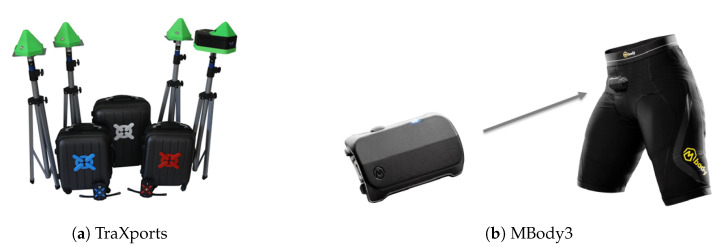
General Overview of the Equipment.

The wearable technology, TraXports, is a system which collects positional data in real-time, allowing the tracking of players during matches and training sessions. This technology is based on ultra-wideband (UWB) technology which provides distance estimation between stationary stations (see Figure 3a) and athletes wearables, without the need for any global positioning system. Only four stationary stations are required to estimate the player’s position, thus minimizing the setup time and complexity of the overall system. TraXports also includes an inertial measurement unit (IMU) that, combined with Kalman filter, improves the player’s position and orientation estimation within the field. The output generated by this equipment can be described and shown above (see Table 2).

- Pos $X_{n}$—X axis position of the *n*^th^ player
- Pos $Y_{n}$—Y axis position of the *n*^th^ player
- Pos $W_{n}$—Angular position of the *n*^th^ player

In addition to TraXports, MBody3 was also considered (see Figure 3b). MBody3 is designed for collecting physiological data due to its capability of measuring the electrical activity of the three major muscles groups in each leg, in real-time. In total, it collects data from 6 muscles as it is shown on Table 3.

### 2.3. Description of Data Extracted

Our data set consists of physiological and kinematic data (position and orientation). Physiological data were collected with a frequency of 25 Hz, sent via Bluetooth BLE 4.0 and then stored on an SD card. As for kinematic data, it was transmitted via UWB wireless technology, with a frequency of 30 Hz, and directly forwarded to a cloud server.

After the data acquisition, applying preprocessing routines was necessary to mitigate hardware failures and limitations. Mbody3 was unable to cope with the needs of on-the-fly data analysis, being unable to provide a constant rate of 25 samples per second, falling below this number during certain occasions. This was tackled by extrapolating the physiological data with a curve-fitting technique, smoothing spline available in Matlab R2018b, making sure that, for every second, 25 new samples were guaranteed. Re-sampling was carried out using the resample function from Matlab, which applies a finite impulse response (FIR) antialiasing lowpass filter to the data and compensates for the delay introduced by the filter. This was applied to TraXports data to guarantee that both systems provide the same number of samples (25 Hz). The last preprocessing phase was the labelling, necessary both for training the methods previously described, as well as to evaluate them with untested data. Labelling was done by hand, using the recorded videos of the games as ground truth.

The classification methods investigated in this article are supervised. Therefore, both labelled training and testing datasets are needed. For the testing dataset, the labelling is mostly needed to compare the result of the models with the expected result (ground-truth label). From the data acquired, it was possible to define seven different actions: (i) running; (ii) running with the ball; (iii) passing; (iv) walking; (v) walking with the ball; (vi) shooting; and (vii) jumping. For this study, three classification methods were adopted: ANN, LSTM and DBMM. None of the events has been eliminated. However, as the trials had highly different sizes, we further investigated the length of the sequences, as the inferior (25%) and the superior (75%) quantiles. There were many trials whose length was inferior to 25 frames (i.e., below 1 s), which made it unfeasible for any of the methods to converge towards the expected action in time. Bearing this in mind, we have eliminated some of the trials, while still ensuring the representativeness of all different actions. Likewise, longer trials were also divided into multiple smaller ones, maintaining a similar average duration of trials, regardless of the action.

### 2.4. Description of the Data Analysis Framework

#### Feature Selection

This section describes the features that were adopted to classify actions using the kinematic and physiological data extracted. In total, there was nine feature, six averaged and normalized EMG signals, velocity, distance to the goal and orientation towards the goal.

1. **Velocity:** This feature relates the variation of the player’s position in the pitch in relation to time and can be calculated as follows:
  (1) $$v=\frac{\sqrt{{\left(x_{t}-x_{t-1}\right)}^{2}+{\left(y_{t}-y_{t-1}\right)}^{2}}}{\Delta t}$$
  where $\left(x_{t},y_{t}\right)$ and $\left(x_{t-1},y_{t-1}\right)$ represents two consecutive positions and Δt the time interval between those points.
2. **Distance to the goal:** The distance is defined by the length of the space between two points. In our case, we computed the distance from a player to the middle line of the opponent goal as:
  (2) $$d=\sqrt{{\left(x1_{t}-x2_{t}\right)}^{2}+{\left(y1_{t}-y2_{t}\right)}^{2}}$$
  where $\left(x_{1},y_{1}\right)$ and $\left(x_{2},y_{2}\right)$ represents the positions of the player and the middle line of the opponent goal, respectively.
3. **Orientation:** The orientation can be defined as the rotation of an object regarding a fixed point and a reference position. For our work, we wanted to determine the orientation of the player towards the middle line of the opponent goal, θ, calculated as follows:
  (3) $$\theta =(W_{t}-atan2\left(\left(y2_{t}-y1_{t}\right),\left(x2_{t}-x1_{t}\right)\right))$$
  where (x2,y2) and (x1,y1) represent the position of the opponent goal and the player, respectively, and *W* is the angular position of the player in the field.
4. **Muscle Activation** As it was described in Section 2.3, the exported EMG data was already filtered (rectified and smoothed) to give an average 25 Hz EMG output, *e*. Smoothing was done by calculating the average values within frame intervals. After that, the features were normalized, by doing:
  (4) $$norm_{e}=\frac{e-min(e)}{max(e)-min(e)}$$
  where $norm_{e}$ is the normalized value of *e*, and max(e) and min(e) represents the maximum and minimum value of all the values, respectively.

The feature selection was carried out before training and testing the model, being that the features used as inputs to the classification methods were equally computed for both training and testing datasets. Then, the features were applied to the entire dataset, processing the raw data into relevant time series to be used as input data. However, the dataset was subsequently divided into two sets, the training set (70% of the data) and the test set (30% of the data), as it can be seen in Table 4, guaranteeing that none of the train data was being used during testing, thus avoiding overfitting.

The dataset is organized into two different ways. For LSTM, the dataset is organized into frames (columns), each comprised of 9 features (rows): six EMG values, velocity, distance to the goal and orientation towards the goals. For ANN and DBMM, the organization of the dataset is inverted. In other words, it is organized into frames (rows), each comprised of 9 features (columns): six EMG values, velocity, distance to the goal and orientation towards the goals.

### 2.5. Description of the Model Trained

#### 2.5.1. ANN

An Artificial neural network (ANN) is a computational system that was inspired by the human brain. This approach is composed of interconnected neurons which are a logical and mathematical structure that behaves like a biological neuron. ANNs can be described as supervised or unsupervised learning methods. In supervised learning, the value of the weights between the neurons are constantly changing (externally) so the output is closer to the expectations. In unsupervised learning, there is no knowledge about the output data. In this case, the algorithm does a class separation by detecting similar features. The network’s learning, in this case, is made by observation. In other words, here the adjust of the weighs is made by the analysis of the input data [24]. In our study, we used a supervised approach where the weights can be adapted in the training phase to improve the performance of the method. More generically, the weights can be updated through the next formula:

(5) $$w_{i}^{t+1}=w_{i}^{t}+\eta e^{t}x_{i}^{t}$$

where η is the learning rate, $x_{i}^{t}$ represents the input data within the instant *t* and $w_{i}^{t}$ is the current weight value. As for $e^{t}$, it represents the error and can be calculated as follows:

(6) $$e^{t}=d^{t}-y^{t}$$

where $d^{t}$ is the expected output and $y^{t}$ is the ANN output [25].

Here, we used the Matlab *Neural Network toolbox* where the hidden layer transfer function is a hyperbolic tangent sigmoid transfer function (tansig), and the output transfer function is a normalized exponential (softmax).

#### 2.5.2. LSTM

Long Short Term Memory (LSTM) network is a sequential classification method, meaning that it takes into consideration the time factor. It is a type of RNN introduced by [23], with the capability of learning long-term dependencies, allowing to remember information for a certain period. This particularity represents an improvement over other RNN models [26,27]. Their chain structure is very similar to typical RNN with the difference that, instead of having a single type of neural network layer. LSTM has four interacting layers, called cell state layer, input gate layer, forget gate layer and output gate layer.

The first step of an LSTM network is to determine which information is going to be thrown away from the cell state ($C_{t}$), and which one passes to the next cell. The decision is, normally, a number between 0 (forget) and 1 (keep), being made by a sigmoid layer, known as the forget gate layer ($f_{t}$):

(7) $$f_{t}=\sigma \left(W_{f}\cdot \left[h_{t-1},x_{t}\right]+b_{f}\right)$$

The result of Equation (7) depends on the previous cell output $h_{t-1}$ and the input vector $x_{t}$. Afterwards, there is the decision step wherein the input gate layer, $i_{t}$, decides which values will be updated:

(8) $$i_{t}=\sigma \left(W_{i}\cdot \left[h_{t-1},x_{t}\right]+b_{i}\right)$$

After that, a tanh layer creates a vector with new values, $\tilde{C}$.

(9) $$\tilde{C}=tanh\left(W_{c}\cdot \left[h_{t-1},x_{t}\right]+b_{c}\right)$$

Then, in the cell unit, the old state is multiplied by $f_{t}$ and then added to $i_{t}$*$\tilde{C}$ to update the cell state:

(10) $$C_{t}=f_{t}*C_{t-1}+i_{t}*\tilde{C}$$

The output gate, *o*, is the vector of output gate activations and it is computed as follows:

(11) $$o_{t}=\sigma \left(W_{o}\cdot \left[h_{t-1},x_{t}\right]+b_{o}\right)$$

where it is decided which parts of the cell state are going to the output. Then, the current cell state is computed with tanh and the result is multiplied by $o_{t}$ so that only the relevant information is provided to the output.

In the equations above, the multiple *W* and *b* represent the respective weight and bias [28,29]. The input weight matrix *W* is a 4-by-NumHiddenUnits matrix and is a concatenation of the weight matrices of the input gate, forget gate, cell candidate and output gate. There are seven ways to initialize the input weights being the Glorot the one adopted in this work [30]. This initializer uses a uniform distribution with mean=zero and $variation=\frac{2}{InputSize+numOut}$, where numOut=4×NumHiddenUnits. The bias *b* is a 4-by-1 numeric vector that, like weighs, is the result of the concatenation of the four bias vectors for the gates. The parameter used to initialize it was the “unit-forget-gate”, which initializes the forget gate bias with ones and the remaining biases with zeros.

#### 2.5.3. DBMM

Ensemble classification methods consist of a set of classifiers combined, offering better results than when using a single classifier. All classifiers in the ensemble predict the correct classification of each unseen instance, and their predictions are then combined using some form of voting system [31]. One such example is the Dynamic Bayesian Mixture Model (DBMM), which combines the likelihoods from multiple classifiers into a single form, attaching different weights to each classifier. According to the posterior probability, it uses an uncertainty measure as a confidence level, which can be updated locally during online classification. This means that the classifier presenting more confidence along the temporal classification is the classifier with more priority [22,32]. To incorporate temporal information as a simple dynamic probabilistic loop, the DBMM assumes a first-order Markov property. The dynamic probabilistic ensemble of classifiers considers the Bayesian probability as a mean of classification, so the result is represented as a weighted sum of the distributions. It should also be noted that DBMM accepts the incorporation of the probability state transition matrix.

The DBMM embraces a set of models $A=\left\{A_{m}^{1},A_{m}^{2},\ldots ,A_{m}^{T}\right\}$, where $A_{m}^{t}$ is a model with *m* spatiotemporal features, at a time instant $t=\left\{1,2,\ldots ,T\right\}$. Therefore, the DBMM general probability distribution function, for each class, *C*, taking the posterior of the previous time instant t−1 as the prior for the present time instant *t*, is:

(12) $$P\left(C^{t}|A\right)=\beta \times P\left(C^{t}|C^{t-1}\right)\times \sum_{i=1}^{N}w_{i}^{t}\times P_{i}\left(A|C^{t}\right),$$

$$\mathrm{with}\left\{\begin{array}{c} P\left(C^{t}|C^{t-1}\right)=\frac{1}{c}\left(uniform\right),t=1\\ P\left(C^{t}|C^{t-1}\right)=P\left(C^{t-1}|A\right),t>1 \end{array}\right.$$

where:

- $\beta =\frac{1}{\sum_{j}\left(P\left(C_{j}^{t}|C_{j}^{t-1}\right)\times \sum_{i=1}^{N}w_{i}^{t}\times P_{i}\left(A|C_{j}^{t}\right)\right)}$ is the normalization factor, necessary due to the continuous updating of the confidence level;
- $P(C^{t}|C^{t-1})$ is the probability distribution of transition between class variables over time;
- The weight $w_{i}^{t}$ is estimated through the level of confidence based on entropy;
- $P_{i}\left(A|C^{t}\right)$ is the *a posteriori* result for the base classifier *i* in the instant of time *t*, which becomes the probability *i* of the mixing model, with $i=\left\{1,...,N\right\}$ being *N* the total number of classifiers considered in the model.

The entropy of the posterior probabilities of each base classifier can be computed to assign the weights $w_{i}^{t}$. Since this algorithm has memory based on its Markov property during the online classification, this also implies that there is temporal information from the set of posteriors for each base classifier. Combined this with the weights at the previous time instant $w_{i}^{t-1}$ to update the weights of each base classifier for current frame classification applying Bayes rules leads to:

(13) $$w_{i}^{t}=P_{i}\left(w_{i}^{t}|h_{i}\right)=\frac{P_{i}\left(h_{i}|w_{i}^{t}\right)\times P_{i}\left(w_{i}^{t-1}\right)}{\sum_{i=1}^{n}P_{i}\left(h_{i}|w_{i}^{t}\right)P_{i}\left(w_{i}^{t-1}\right)}$$

$$\mathrm{with}\;i=\left\{1,2,\ldots ,n\right\}$$

In order to obtain $P_{i}\left(h_{i}|w_{i}^{t}\right)$, Equation (12) is computed over the new set of posteriors from each base classifier from previous posteriors of the test set.

In this work, the base classifiers adopted are Naive Bayes (NB), Artificial Neural Network (ANN) and k-Nearest Neighbors (k-NN). The NB assumes that features are independent of each other given the class variable. Thus, one probability density function (PDF) for each feature model was considered, obtaining the following expression:

(14) $$P\left(C_{i}|A\right)=\alpha P\left(C_{i}\right)\prod_{j=1}^{m}P\left(A_{j}|C_{i}\right)$$

where $\alpha =\frac{1}{\sum_{i}P\left(A|C_{j}\right)P\left(C_{i}\right)}$ is a normalization factor and *m* is the number of independent feature models. The inference is usually achieved by using the maximum a posteriori (MAP) estimation [11]. The ANN can learn complex nonlinear input-output relationships. It is a logical and mathematical structure that consists of inputs, which are further multiplied by a weight determining the activation of the neuron, all the way until it reaches the last layer (i.e., output layer) [33]. Here, the hidden layer transfers function is a hyperbolic tangent sigmoid and the output transfer function is a normalized exponential. At last, k-NN is incorporated by objects that are classified based on a similarity measure, such as the Euclidean distances. This classifier learns from the training patterns and adapts itself to recognize or classify the unknowns test patterns. Despite the simplicity of this method, it is vastly used in pattern recognition [3].

### 2.6. Evaluation Metrics

To assess the performance of both LSTM and DBMM, we considered the following traditional metrics:

(15) $$Accuracy=\frac{TP+TN}{TP+TN+FP+FN}$$

(16) $$Precision=\frac{TP}{TP+FP}$$

(17) $$Recall=\frac{TP}{TP+FN}$$

(18) $$F1Score=2*\frac{Precision*Recall}{Precision+Recall}$$

where *TP* = True Positives, *TN* = True Negatives, *FP* = False Positives and *FN* = False Negatives.

For our specific case, F1 Score is the most suitable evaluation metric as it has been used in cases where data sizes are unbalanced. This metric calculates the relation between precision and recall. Therefore, if the result of F1 score is high, both precision and recall of the classifier indicate good results, as it normally emphasizes the lower value https://towardsdatascience.com/accuracy-precision-recall-or-f1-331fb37c5cb9.

## 3. Experimental Results

The current Section shows the results of both action recognition approaches. This section starts by using the LSTM to evaluate the impact of feature selection in the classification result.

### 3.1. Evaluating Features

To establish the set of features with the best score, we did some tests adding or removing some of the features described in Section 2.4. This study was composed of five tests, divided as: (*i*) EMG features; (*ii*) EMG + velocity; (*iii*) EMG + distance to goal; (*iv*) EMG + Orientation towards the goal feature; and (*v*) All features(EMG + velocity + distance to the goal + orientation towards the goal).

**Figure 4:**
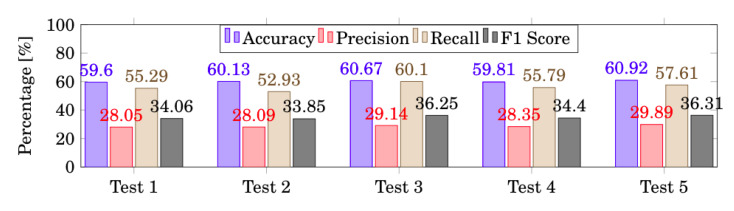
Evaluation Metrics per Test.Evaluation Metrics per Test.

Figure 4 depicts the behaviour of the model, for each test, according to each evaluation metric. As stated before, more emphasis should be given to the F1 score due to the inconsistent number of trials for each action. This led us to conclude that the fifth test is the one with better results achieved, implying that all features should be considered for further analysis and to compare ANN, LSTM and DBMM approaches.

It was needed to study the number of features to use as it is not generic that more features mean better performance. As an example of that, we have the study made by Sabeti et al. [34], where they wanted to determine the relevant features for classifying schizophrenia. In this study, they employed a genetic algorithm to select the best features, from all the channels, to reduce the complexity and redundancy made by feature selection. To test it was used two classification methods LDA (Linear discriminant analysis) and SVM. Contrarily to the result of our study, while using all the features based on each channel, the accuracy was lower than when using features from channels with a better discrimination ability. One of the feature extraction methods that was used was the Autoregressive (AR) coefficients. For this method, and using all the features extracted, the mean accuracy was 66.62% and 70.56% for LDA and SVM, respectively. However, when using only the features that showed a better discriminant ability with a bidirectional technique, they obtained 83.15% and 99.31% for LDA and SVM, respectively.

### 3.2. Benchmark

The ANN is a widely used method in the literature, reason why it was used to test our dataset and compare with not as classic methods as LSTM and DBMM. Even though being widely used in the literature we were unable to achieve good results while using ANN (see Table 5). Then, we tested the data with LSTM and DBMM. However, although ANN does not present good results when used as a single classifier, when combined with other classifiers, it attains a superior overall performance as it can be seen in DBMM results. To train and test the model more diversely, we repeated the process of splitting the data into training (70%) and test (30%), thirty times, randomly. All three confusion matrices and metrics contains the average ± standard deviation, (Table 5, Table 6, Table 7, Table 8, Table 9 and Table 10). The confusion matrices also contains the total percentage, gathering all the features in the study, for the thirty tests.

Table 5 shows the performance of the ANN approach. We can see that the action with the higher true positive (TP) value obtained was “Walking”. In terms of the worst classification value, the classes with the lowest percentage of correct classifications were “Shooting”, “Walking with the ball”, and “Jumping”, respectively.

According to the overall results, focusing on the F1 score, “Walking” with 78.74% of F1 score, was the only action whose metric value was superior to 20% as it can be seen in Table 6. “Walking with the ball” had an F1 score value of about 1% (1.02%). Globally, the ANN approach had 90.03% of accuracy, 16.06% of Precision, 67.87% of recall and an F1 score of 14.72%. But, besides the metrics presented, what stands out in the behaviour of the ANN is the fact that all classes are mostly classified as “Walking”, the action with the highest number of samples present in our data set.

As for LSTM, the Table 7 shows that, as well as in ANN, the action with the higher true positive (TP) value obtained was “Walking”.

The classes with the lowest percentage of correct classifications were “Walking with the ball”, “Shooting” and “Jumping”, respectively. According to the overall results, focusing on the F1 score, the actions “Passing” and “Walking” had an overall F1 score of 71.89% and 73.80%, respectively, as it can be seen in Table 8. “Walking with the ball” had an F1 score value lower than 1% (0.82%). Globally, the LSTM approach had 60.92% of accuracy, 29.89% of Precision, 57.61% of recall and an F1 score of 36.31%. Although it does not perform well for all the actions, the LSTM can recognise and correctly classify some of the actions, such as walking and passing.

Table 9 presents the confusion matrix with the results obtained using DBMM. “Walking” was the action with the highest success rate, having 194.70 well-classified trials in a total of 207, followed by “Walking with the ball” with 6.79 trials in a total of 8. “Running” and “Jumping” also had good results, however, in about 16% of the time, those actions were misclassified as “Walking”, a situation that happened with all of the other actions. However, all the actions had a success rate higher than 65%, being “Passing” the one with the lowest results having only 15.98 well-classified events in a total of 24.

Table 10 shows the results of accuracy, precision, recall and F1 score. “Walking” is the most recognizable action, having an F1 Score of 92.28%, a recall of 90.56% and a precision of 94.06%. “Walking with the ball”, “Jumping” and “Running” belongs to the group of actions with better results. “Passing” and “shooting” deserve to be highlighted for their recall, having 86% and 84.16%, respectively. The remaining actions, despite presenting fairly high accuracy values, are the ones with the lowest results in the other metrics. Nevertheless, the good performance of DBMM is easily recognized, having achieved a global accuracy of 96.47%, a precision of 77.70%, a recall of 84.12%, and an F1 score of 80.54%.

As ANN did not fulfil the expectations, a final comparison was made only between LSTM and DBMM that fell on the computation resources needed to classify actions. Both approaches were deployed in the same computer of 2.50 GHz, 8 GB RAM, with a GeForce GTX 1080. As we can see in Table 11, besides the better results achieved with DBMM, the elapsed time occurred between the training and the results is higher than when using the LSTM network approach. The LSTM testing time is 0.236 s and for DBMM, it is 0.382 s.

### 3.3. Discussion

This research paper presents three supervised methods to recognize actions from athletes using EMG and positional data obtained by combining Mbody3 with TraXports. During acquisitions, both systems were collecting and recording data for subsequent analysis. Six EMG channels collected physiological data, specifically right and left quadriceps, hamstrings and gluteus, while TraXports collected the position and orientation of players in the field ($x_{t}$, $y_{t}$, $\theta_{t}$). Seven actions were selected to be recognized by the methods: walking, walking with the ball, running, running with the ball, shooting, passing and jumping. While some actions were well represented in the dataset, others were not, with less than 20 representative trials for training (see Table 4). This is the reason that justifies the behaviour observed in the ANN, where the discrepancy’s present between the different classes is evident, making the learning process of the algorithm and its consequent decision making very much influenced by the class that presents the most data. This also could be one of the reasons why the LSTM network presented an inferior performance since it is known that LSTM, as a deep learning approach, requires a large representative dataset.

Concerning the data features, the EMG data were always included since all the actions performed involved the lower limbs. This was combined with other features to attempt in distinguishing certain actions, such as those with the presence and absence of the ball. For example, in LSTM, it was found that 1.79% of the actions referring to “Walking” are confused with “Walking with the ball”. In this case, the velocity can make all the difference in the recognition of these actions, as well as the orientation and the distance to the goal, from where it is verified if the player is doing an offensive or a defensive action, giving all these features a great contribution to the classification. By including the velocity as a feature, it was possible to improve the accuracy from 59.6% to 60.13% (Figure 4). By including the distance to the goal, we also got even better results, improving the accuracy from 59.6% to 60.67% (Figure 4). This is an expected improvement since this feature can give us the perception of offensive or defensive actions, which can be an important indicator to distinguish between shooting and passing. As for the orientation to the goal, we have not noticed much improvement, because this feature, when used independently, does not provide much information. The last test, where all the different features have been combined, is the one leading to better results. All the classification methods depend on the quantity and quality of the data, so having a whole set of complementary features allow achieving better outcomes.

After analysing and comparing Table 6, Table 8 and Table 10, we concluded that both ANN and LSTM model were not the most reliable choice to identify the actions that were proposed for the existing dataset. For “Walking”, as the action with the largest number of trials available, ANN and LSTM had an F1 score of 78.74% and 73.80%, respectively, suggesting that its performance highly relies on the number of trials in the dataset. Still, “Walking” was also the activity leading to better results using DBMM, with an F1 Score of 92.28%. Regarding the lowest F1 Score, the methods led to different results. With ANN, the lowest classified action was shooting with an F1 score of 0.43%. Using LSTM, the worst, in terms of classification, was “Walking with the ball” (0.82%). In DBMM, the action with the lower result was “Running with the ball” (73.75%). “Shooting”, despite occupying only 1% of the dataset, still managed to have a recall of 100% on ANN and approximately 84% on DBMM. However, the recall fell to 43.30% when using the LSTM. “Jumping”, corresponding to 2% of the data, was the fourth activity with the greatest success on DBMM, having an F1 Score of approximately 83%. The same action, however, had an F1 score of 30.38% using the LSTM and even worst on ANN (1.95%). As opposed, “passing” was the second most recognized action by the LSTM, occupying the fifth position for the DBMM, with a recall of 86%. As for ANN, this action had an F1 score of 1.95% due to its low precision (0.99%). It should be noted that the DBMM still outperforms the LSTM in terms of precision, accuracy and F1 Score for all actions. The same happened with recall, except for the “passing” action, which in LSTM is approximately 93%. ANN outperforms both LSTM and DBMM in terms of “walking” precision and also in “shooting” recall.

Taking a more general view, the global values of the implemented metrics presented in Table 12, indicates that DBMM has a higher success rate when comparing with LSTM, being also the faster training algorithm.

With these results, we can state that action recognition can be achieved when combining wearable devices and an artificial intelligence method, being the method selection, its parameterization, and feature selection, critical steps in the result. Although ANN is one of the most widely used methods in the literature, when tested alone, it couldn’t give the expected results. However, when combined with another classification method, as it is the case in DBMM, the combined solution can achieve a higher classification performance.

## 4. Conclusions and Future Work

This research paper focused on the study of action classification in sports using wearable technology combined with a deep learning method (i.e., LSTM) and an ensemble classification method (i.e., DBMM), as well as comparing these two methods with the ANN, which is a classification method with a long history in the literature. To do so, we developed an architecture for data integration coming from two commercial solutions: TraXports and MBody3. After that we proceed to data acquisition, preprocessing and, lastly, we trained and tested the models so they could classify actions as passing, shooting, running with and without the ball, walking with and without the ball and jumping.

According to the literature and also with our results, we can say that feature selection is an important step for the pattern recognition study. That allied to an aware choice of the classification method, as well as its parameterization, the overall classification performance can be improved.

For future work, this study should benefit from augmenting the dataset by providing more trials. This could be the major contributing factor for the LSTM and ANN methods. A convergence analysis between methods should also be conducted. Since the system is intended for a real-time application, it is of the utmost importance to evaluate which of the two classifiers converges to the adequate action the fastest.

## Figures and Tables

**Table 1:** Target Actions.

Target Actions						
Walking	Walking with ball	Running	Running with ball	shooting	Passing	Jumping

**Table 2:** Example of TraXports CSV file.

TraXports Example File				
idx	posX1	posY1	posW1	...
1	0	0	100	...

**Table 3:** Example of Mbody3 CSV file.

MBody3 Example File						
R.Quad	L.Quad	R.Hams	L.Hams	R.Gluteo	L.Gluteo	Time(s)
45	35	21	2	11	3	0.04

**Table 4:** Entire Dataset.

Actions	*Number of Trials*
Running	183
Running w/ball	57
Passing	80
Walking	690
Walking w/ball	27
shooting	15
Jumping	24
70% of the Dataset.	
**Actions**	***Number of Trials***
Running	128
Running w/ball	40
Passing	56
Walking	483
Walking w/ball	19
shooting	11
Jumping	17
30% of the Dataset.	
**Actions**	***Number of Trials***
Running	55
Running w/ball	17
Passing	24
Walking	207
Walking w/ball	08
shooting	04
Jumping	07

**Table 5:** ANN Confusion Matrix.

**Target Class**	Running	**6.38 ± 1.09** **1.98%**	0.02 ± 0.03 0.01%	0.01±0.01 0.001%	48.58 ± 1.11 15.09%	0.01 ± 0.01 0.003%	0 ± 0 0%	0.01 ± 0.01 0.002%
	Running w/ball	1.67 ± 0.15 0.52%	**0.10± 0.03** **0.03%**	0 ± 0 0%	15.22 ± 0.18 4.73%	0.01± 0.01 0.001%	0 ± 0 0%	0.01 ± 0.01 0.002%
	Passing	1.04 ± 0.22 0.32%	0.04 ± 0.05 0.01%	**0.17 ± 0.08** **0.05%**	22.79 ± 0.23 7.08%	0.01 ±0.02 0.004%	0 ± 0 0%	0 ± 0 0%
	Walking	4.37 ± 0.24 1.36%	0.04 ± 0.03 0.01%	0.02 ± 0.08 0.01%	**202.54 ± 0.29** **62.90%**	0.02 ± 0.02 0.01%	0 ± 0 0%	0.01 ± 0.01 0.003%
	Walking w/ball	0.23 ± 0.12 0.07%	0 ± 0 0%	0 ± 0 0%	7.73 ± 0.16 2.40%	**0.04 ± 0.03** **0.01%**	0 ± 0 0%	0 ± 0 0%
	Shoting	0.18 ± 0.12 0.06%	0 ± 0 0%	0 ± 0 0%	3.81 ± 0.13 1.18%	0 ± 0 0%	**0.01 ± 0.01** **0.003%**	0 ± 0 0%
	Jumping	0.10 ± 0.02 0.03%	0.01 ± 0.01 0.003%	0 ± 0 0%	6.82 ± 0.06 2.12%	0 ± 0 0%	0 ± 0 0%	**0.07 ± 0.03** **0.02%**
		Running	Running w/ball	Passing	Walking	Walking w/ball	Shoting	Jumping
	**Output Class**							

**Table 6:** ANN Evaluation Metrics.

Actions	*Accuracy*	*Precision*	*Recall*	*F*1*Score*
Running	82.73 ± 0.8253	11.60 ± 0.48	45.63 ± 0.55	18.50 ± 0.51
Running w/ball	94.72 ± 0.90	0.58 ± 0.09	49.10 ± 0.20	1.15 ± 0.12
Passing	92.58 ± 0.88	0.70 ± 0.13	86.03 ± 0.80	1.38 ± 0.22
Walking	66.02 ± 0.52	97.85 ± 0.48	65.87 ± 0.13	78.74 ± 0.21
Walking w/ball	97.51 ± 0.92	0.52 ± 0.10	45.07 ± 0.36	1.02 ± 0.16
shooting	98.76 ± 0.95	0.22 ± 0.05	100 ± 1	0.43 ± 0.09
Jumping	97.84 ± 0.97	0.99 ± 0.21	83.38 ± 0.57	1.95 ± 0.31
**Total**	90.03 ± 0.85	16.06 ± 0.22	67.87 ± 0.52	14.74 ± 0.23

**Table 7:** LSTM Confusion Matrix.

**Target Class**	Running	**11.07 ± 2.76** **3.44%**	2.03 ± 1.43 0.63%	1.80 ± 0.98 0.06%	38.20 ± 3.15 11.86%	0.57 ± 0.62 0.18%	00.70 ± 0.82 0.22%	0.63 ± 0.87 0.20%
	Running w/ball	3.07 ± 1.41 0.95%	**1.43 ± 1.05** **0.45%**	0.60 ± 0.71 0.19%	9.87 ± 1.84 3.06%	0.43 ± 0.72 0.14%	1.10 ± 1.11 0.34%	0.50 ± 0.67 0.16%
	Passing	2.43 ± 1.36 0.76%	1.63 ± 1.35 0.51%	**10.93 ± 2.53** **3.40%**	1.83 ± 1.75 0.57%	1.73 ± 1.36 0.54%	3.40 ± 1.40 1.06%	2.03 ± 1.11 0.63%
	Walking	18.17 ± 3.85 5.64%	2.40 ± 1.70 0.75%	1.60 ± 1.17 0.50%	**182.20 ± 4.97** **56.58%**	1.00 ± 1.21 0.31%	0.83 ± 0.97 0.26%	0.80 ± 0.79 0.25%
	Walking w/ball	0.73 ± 0.93 0.23%	0.33 ± 0.47 0.10%	0.57 ± 0.72 1.18%	5.77 ± 1.05 1.79%	**0.03 ± 0.18** **0.01%**	0.43 ± 0.50 0.14%	0.13 ± 0.34 0.04%
	Shoting	0.43 ± 0.50 0.14%	0.83 ± 0.86 0.26%	1.40 ± 0.84 0.44%	0.33 ± 0.65 1.10%	0.17 ± 0.37 0.05%	**0.50 ± 0.56** **0.16%**	0.33 ± 0.47 0.10%
	Jumping	0.77 ± 0.80 0.24%	0.80 ± 0.83 0.25%	1.60 ± 1.11 0.50%	1.67 ± 1.11 0.52%	0.33 ± 0.60 0.10%	0.83 ± 0.90 0.26%	**1.00 ± 0.77** **0.31%**
		Running	Running w/ball	Passing	Walking	Walking w/ball	Shoting	Jumping
	**Output Class**							

**Table 8:** LSTM Evaluation Metrics.

Actions	*Accuracy*	*Precision*	*Recall*	*F*1*Score*
Running	57.45 ± 0.38	30.30 ± 0.62	65.62 ± 0.59	41.31 ± 0.67
Running w/ball	55.07 ± 0.29	15.11 ± 0.56	58.91 ± 3.67	23.41 ± 1.12
Passing	77.28 ± 0.27	58.86 ± 0.37	93.01 ± 0.32	**71.89**± 0.41
Walking	72.99 ± 0.22	76.00 ± 0.11	71.78 ± 0.34	**73.80**± 0.13
Walking w/ball	50.61 ± 0.34	—	2.88 ± 0.53	**0.82**± 0.15
shooting	53.42 ± 0.74	7.95 ± 1.45	43.30 ± 7.90	12.56 ± 2.29
Jumping	59.59 ± 2.70	21.01 ± 5.28	67.74 ± 5.44	30.38 ± 6.52
**Total**	60.92 ± 0.70	29.89 ± 1.20	57.61 ± 2.68	36.31 ± 1.61

**Table 9:** DBMM Confusion Matrix.

**Target Class**	Running	**44.42 ± 0.69** **13.79%**	0.90 ± 0.18 0.28%	0.57±0.13 0.18%	8.64 ± 0.67 2.68%	0.23 ± 0.11 0.07%	0.11 ± 0.07 0.03%	0.13 ± 0.09 0.04%
	Running w/ball	1.11 ± 0.81 0.34%	**12.28± 1.34** **3.81%**	0.28 ± 0.31 0.09%	3.12 ± 1.06 0.97%	0.09 ± 0.22 0.03%	0.09 ± 0.18 0.03%	0.04 ± 0.12 0.01%
	Passing	1.44 ± 0.94 0.44%	0.63 ± 0.67 0.20%	**15.98 ± 2.11** **4.96%**	5.60 ± 1.68 1.74%	0.24 ±0.25 0.07%	0.03 ± 0.10 0.01%	0.09 ± 0.16 0.03%
	Walking	6.99 ± 0.12 2.17%	2.22 ± 0.07 0.69%	1.55 ± 0.08 0.48%	**194.70 ± 0.18** **60.47%**	0.76 ± 0.04 0.24%	0.33 ± 0.03 0.10%	0.46 ± 0.04 0.14%
	Walking w/ball	0.19 ± 0.79 0.06%	0.08 ± 0.43 0.02%	0.08 ± 0.36 0.03%	0.84 ± 0.97 0.26%	**6.79 ± 1.15** **2.11%**	0.01 ± 0.08 0.002%	0.01 ± 0.18 0.004%
	Shoting	0.27 ± 1.28 0.08%	0.15 ± 1.17 0.05%	0.04 ± 0.68 0.01%	0.84 ± 0.97 0.26%	0.01 ± 0.58 0.005%	**2.67 ± 3.89** **0.83%**	0.02 ± 0.56 0.01%
	Jumping	0.23 ± 1.09 0.07%	0.04 ± 0.41 0.01%	0.07 ± 0.62 0.02%	1.11 ± 2.18 0.34%	0.04 ± 0.40 0.01%	0.002 ± 0 0.001%	**5.51 ± 2.73** **1.71%**
		Running	Running w/ball	Passing	Walking	Walking w/ball	Shoting	Jumping
	**Output Class**							

**Table 10:** DBMM Evaluation Metrics.

Actions	*Accuracy*	*Precision*	*Recall*	*F*1*Score*
Running	93.54 ± 0.82	80.18 ± 0.36	81.18 ± 0.12	80.97 ± 0.18
Running w/ball	97.28 ± 0.09	72.21 ± 0.20	75.35 ± 0.04	73.75 ± 0.07
Passing	96.71 ± 0.83	66.56 ± 0.16	86.00 ± 0.03	75.04 ± 0.05
Walking	89.91 ± 0.71	94.06 ± 0.22	90.56 ± 0.07	92.28 ± 0.10
Walking w/ball	99.20 ± 0.88	84.82 ± 0.20	83.39 ± 0.04	84.10 ± 0.07
shooting	99.36 ± 0.77	66.75 ± 0.11	84.16 ± 0.02	74.45 ± 0.03
Jumping	99.29 ± 0.84	78.75 ± 0.15	88.22 ± 0.02	83.22 ± 0.04
**Total**	96.47 ± 0.71	77.70 ± 0.20	84.12 ± 0.05	80.54 ± 0.08

**Table 11:** Elapsed time for Training and Testing.

Approaches	*Train [s]*	*Test [s]*
LSTM	382.515	0.236
DBMM	29.397	0.382

**Table 12:** Global evaluation metrics: ANN vs LSTM vs DBMM.

Algorithm	*Accuracy*	*Precision*	*Recall*	*F*1*Score*
ANN	90.03 ± 0.85	16.06 ± 0.22	67.87 ± 0.52	14.74 ± 0.23
LSTM	60.92 ± 0.70	29.89 ± 1.20	57.61 ± 2.68	36.31 ± 1.61
DBMM	96.47 ± 0.71	77.70 ± 0.20	84.12 ± 0.05	80.54 ± 0.08
**Total**	96.47 ± 0.71	77.70 ± 0.20	84.12 ± 0.05	80.54 ± 0.08
